# Supplementary material for: Hemimetabolous insects elucidate the origin of sexual development via alternative splicing
Source: eLife. 2019 Sep 3;8:e47490. doi: 10.7554/eLife.47490 (PMC6721801; doi:10.7554/eLife.47490)
Supplement: Supplementary file 4. [file elife-47490-supp4.rtf]

Supplemental File 4: Queries used in BLASTP searches of arthropod gene models for DMRT homologs. 

>gi|17986183|ref|NP_524428.1| doublesex-Mab related 93B [Drosophila
melanogaster]
MASSSGEPADEVANKRPRLVANPKATKIVEPTPAKVTNRVPKCARCRNHGIISELRGHKKLCTYKNCKCAKCVLIFERQRIMAAQVALKRQQAVEDAIAMRLVANKTGRSIDALPPGNIFGLTVTQPSSPRAKREDDQVEKTISEVEQQHLKQREEEYSESPAVAQDLSAPRRDNVSQTAIDMLAQLFPQRKRSVLELVLKRCDLDLIRAIENVSPTGKSSPVDVTSNQLPSQEPGMLTTMPQPAPPRSSAFRPVITDQYQSKSVVELKPPVFGGSASAAAAAAICAYPKWFVPLSFPVTMGHLSNLAPRCTLPNCACLDSYQFH
>gi|17986191|ref|NP_524549.1| doublesex-Mab related 99B [Drosophila
melanogaster]
MSLPSGVDMQNLMSQHPVLGALPPAFFLRAASERYQRTPKCARCRNHGVVSALKGHKRYCRWRDCVCAKCTLIAERQRVMAAQVALRRQQAQEENEARELGLLYTSVPGQQNGSDSATPTPHSPNHSGSGGSGSGGSGQNGVFHGGIPSPPSDGFEASSTPNHHQQSQQQQQAHHQQHLSHPHQQSQRFNGNESDTDGRTRSEHLSVGFSPTRTELDESPVSKRGAALSNETDQDTGSESGSPSSPRPKVAGLFNLTASLSPARTGPPSSPESDLDVDSAPDEATPENLSLKKEDSQSPPNTPAENLHLLRSFSSSHAQGFLPYHHTQFLAAAGLPAHHHPAAHSPHHQQQQQQQQQQQNHLPQHHQQQQQQQQQQQRSPIDVLMRVFPNRRRSDVEQLMQRFRGDVLQAMECMLAGEDLGQTPPQVPPSPPFPMKSAFSPLVPPSVFGSPTHRYHPFMQAHAKRFLTAPYAGTGYLPGVLSAADIEQSESNGAGGIGLDRTSNAGDSQD
>gi|475973|gb|AAA17840.1| doublesex, partial [Drosophila melanogaster]
MVSEENWNSDTMSDSDMIDSKNDVCGGASSSSGSSISPRTPPNCARCRNHGLKITLKGHKRYCKFRYCTCEKCRLTADRQRVMALQTALRRAQAQDEQRALHMHEVPPANPAATTLLSHHHHVAAPAHVHAHHVHAHHAHGGHHSHHGHVLHHQQAAAAAAAAPSAPASHLGGSSTAASSIHGHAHAHHVHMAAAAAASVAQHQHQSHPHSHHHHHQNHHQHPHQQPATQTALRSPPHSDHGGSVGPATSSSGGGAPSSSNAAAATSSNGSSGGGGGGGGGSSGGGAGGGRSSGTSVITSADHHMTTVPTPAQSLEGSCDSSSPSPSSTSGAAILPISVSVNRKNGANVPLGQDVFLDYCQKLLEKFRYPWELMPLMYVILKDADANIEEASRRIEE
>gi|24641758|ref|NP_511146.2| doublesex-Mab related 11E [Drosophila
melanogaster]
MHFDETRSPNKDVRIGKSQRLAANGRQGTDPESDPLHARCVVPHASTPRGRRIPWGHNDDDDDGNHLTLPDSLTATSSCGCQCLNVRAPFHILLVDQQARMSKDASVRICQRERRLLRTPKCARCRNHGVISCVKGHKRLCRWRECCCPNCQLVVDRQRVMAAQVALRRQQTMEALEATASSTKSTGVTTASANNSSSSEGEDSLSSTSPPPAHSHPHSHSHPTSCVSNSSSSSATRQALMAQKRIYKQRLRSLQQSTLHITAAMEEYKQRFPTFSSPLMERMRKRRAFADPELNHVMEATLGGNALYFATVAAAAAACAPVHQEQHIYHPMPPTIPLTIPPTNPAMTTPAVTTASTGSGKKPKLSFSIESIMGIST
>gi|201023333|ref|NP_001128407.1| doublesex isoform F2 [Apis mellifera]
MYREENEQNRAADLAPQQPSGANTFERLEHSQDSKNGDDGSKKVQTDASSSTNTPKPRARNCARCLNHRLEITLKSHKRYCKYRTCTCEKCKITANRQQVMRQNMKLKRHLAQDKVKVRVAEEVDPLPFGVENTISSVPQPPRSLEGSYDSSSGDSPVSSHSSNGIHTGFGGSIITIPPTRKLPPLHPHTAMVTHLPQTLTSENVEILLEHSSKLVELFQYPWEALLLMYINLKYAGANPEEVVRRMVDALIIFCSKNFIWNSILNKIVSFINLLPT
>gi|328781748|ref|XP_003250026.1| PREDICTED: uncharacterized protein
LOC100577410 [Apis mellifera]
MKTENNNSTQVNSGIEQRLRSPKCARCRNHGVISGLKGHKKSCAWKDCRCPCCLLVVERQRVMAAQVALRRQQQAQGNFFSENVSTVCHAPIDTAGSSYFKTDQTPICRRGKNIQRHQLHFTQTSISVTQGFYGLKTIHDLPTAFTADAPLLNGMSQNQEYKQESEDSKKIQSFPSMYSTIPWFEQVTEPRKMKKSFNNFTSAVNDHNDISVAKNRSCLEKKSTISFSVESIIGTK
>gi|328783782|ref|XP_392966.4| PREDICTED: doublesex- and mab-3-related
transcription factor A2-like [Apis mellifera]
MLRGKSKKVENEETEGELKQRRPKCARCRNHGLISWLRGHKRECRYRECLCPKCSLIAERQRVMAAQVALKRQQAAEDAIALKMAKVATGQKLSRLPPGKIFGMAVTDPKSVGDSNKQENPPSKEDLGDEDPKKDDSLNDQCLNSSVCDHIESIPKNKNLVFNVNNREETKESSVSQTSVETLARLFPNTKLSVLQLVLQRCGQDLLKAIEYFASDSFGINSTTYTSAFQPPQSINETRSNEQIAGTMLAPIYSSLSRNFYGDGYCFLNIVPEQFPNSIVDATSCTALPIKNSGHEQEGVALNVQYNNYFNSGTQQQLRDHVYTQVTDHLSPRPSFLHLPSVLSGIPCVQPNCSQCIYKFP
>gi|11967930|dbj|BAB19780.1| Bmdsx [Bombyx mori]
MVSMGSWKRRVPDDCEERSEPGASSSGVPRAPPNCARCRNHRLKIELKGHKRYCKYQHCTCEKCRLTADRQRVMAKQTAIRRAQAQDEARARALELGIQPPGLELDRPVPPVVKAPRSPMIPPSAPRSLGSASCDSVPGSPGVSPYAPPPSVPPPPTMPPLIPPPQPPVPSETLVENCHRLLEKFHYSWEMMPLVLVIMNYARSDLDEASRKIYEGKMIVDEYARKHNLNVFDGLELRNSTRQKMLEINNISGVLSSSMKLFCE
>gi|512930282|ref|XP_004932028.1| PREDICTED: doublesex- and mab-3-
related transcription factor A2-like [Bombyx mori]
MLNNSGRARVPKCARCRNHGLISSLRGHKKACAYRHCQCPKCGLIKERQRIMAAQVALKRQQAAEDKIALHLASVETGTPLESLPPGRIYGMRVTDPSPSPGPEPDSAADDQIPIHIDSETSDSLPDCSNVSPDSVGNSSGVSQLRSGQDESTVEAEGAVSTAGLEMLRKLFPGKKRSVLELVLRRCNHDLLRAVEHFNATHGRDKIPESTTGLGFEASVSSSEDPESRWSAFRPVSRRAPLLPALVMGRVYGSEWLVPLPALPTLSTPLLLPLQHQHVPPASCNPCAPDCRQCNNHH
>gi|512923084|ref|XP_004930266.1| PREDICTED: doublesex- and mab-3-
related transcription factor 2-like [Bombyx mori]
MQSEISENNNSTRKALRTPKCARCRNHGVISCLKGHKRLCRWRDCRCPGCLLVLERQRVMAAQVALRRQQGAGGPESRNSEAAALAARKRAYRARLRCMQMSRTYTVQPPNFGGNEILFPNDPAWNERVRRRKAFADAELERGVGGTNMAAPPAALLCRNLLAALLTTYQPAVQVPPQRQKLSFSIESIIGVQ
>gi|169807984|dbj|BAG12872.1| doublesex-Mab related 93B [Daphnia magna]
METSNSSKSAMSINNGSASCSSSGGAALRRPKCARCRNHGVISWLKGHKRHC
RFKDCLCVKCNLIAERQRVMAAQVALKRQQATEDAIALGLRSVATGTRMPFLPPGPIFGGRDSPKKEDTLDESMYNSGSDDEELQAIGQEHQQRTVEKVLNLALNNNDDPEISESPAGTNKERILQHHGSLDMLTRVFPFHPKNAVESALENCGGDVAKAVQQLVGHQPNHQSSSTDIVMMTNAEDGTMVTSSKANNGGSNYLMTTSGSNKSAFMPTTSLLSVPEISSPNRSQYGHQMSASVSSSTSMAYPSALRMMPPYPSAAGMMSFLHPSAYFAAAASAAAAAASNPHSYPWLFSSHYRPPSQHHQFQHICLPGCSVCPISTTSGTSSTSSSSSPGEGVGCNPNNSNV
KTSTLMLSSSPDRLFKTNIGRNKDLLFQHSSGGNNFP
>gi|321463769|gb|EFX74782.1| DM DNA-binding protein [Daphnia pulex]
MDGGGGSSSPLRFGVKKMAEAVGGGKQRLLRTPKCARCRNHGVVSCLKGHKKLCRWKECQCTNCLLVVERQRVMAAQVALRRQQNSESGKGDGGLNNKQPSKVTKSAEAILAQKKLYQKHLRTLQQSSLARDVLQNYRQWVQRGHNGSAISPEGGDQQQNSDKISVTPPPPLSERMRKRRAFADRDLDAVMFQREQQAAVELQRFNISPGCGGDATPPPSSLGPAPTPCWNALHPIRFHVLPFHQHLIQQQQTHPLCPLPLVVKTEAKPSDSDDSDVEVDVTSTPSTPPSPILLPSVSSSSTNKSKISFSVESIIGRR
>gi|321468109|gb|EFX79096.1| Doublesex and mab-3 related transcription
factor-like protein 1 [Daphnia pulex]
MPFSDEESSEMLSPSSQTQFDHSETIDVEHMDEDELKSSPNSFGCAGKGASCRNPTCALCKNHGINSPLKGHKRYCPFGRCSCDLCRVTRKKQKINASQVATRRAQQQDRELGIDRPTMQTNTPSGSSTPRTFASSASPSDLGRNSTRSALDVERPLIEPNPTQYIPSHYTPGSGPEEFGKNMALECFLLGRRASLLVNSLRDSRLTMEHLAYLDNDVCTAICIIRDEVSTQLNDIYQYLIDRLQREQISNVTAKSNAAATVPSYIANQFAILSEQSSRVASEEAFSVSQLYGHSSRDTVICPPRHLSLPVYPYVNQ
>gi|321473901|gb|EFX84867.1| DMRT-99B-like protein [Daphnia pulex]
MDLSSHRNSGHQVPSGDGSGGSGGPGSASNGMMSPHGFFAGLAGHMGSPHQHHGHHHQQHHGQQHHPGHHHHHHGGIPPTAAAAAAAMLLRASERYQRTPKCARCRNHGVVSALKGHKRYCRWRDCACAKCTLIAERQRVMAAQVALRRQQAQEENEARELGLLYTVPSSGMNNMGQQAGPQGPAGQDSPTRSMSDRSGSGGGRKSANRAESGCDSSANNGYDNSVNKRLNYSGREAGQHPPGPLPPICPTGSERDESSHQHYHPYMNRKGDGLLNTAEPLSPPTNRASKMSNNSLSPKSPQLMDSDSDDAGDSDHNSHPTDEPELLLPSDRTKFELADVDDNGSALEAASGGGSEPEAASKRVPLDTLARLFPQTKRATLKSTLDRCDGDVLRAIEQLVYHNNNPQPEGNNGSTVNANEANLEGAHPASGNSNQHKRKSSEHSSSGSREKHVPRYNHHHHHHQHHPHPQAAAEASLQWKNCLSAVGNAFPNAGSLGGGSSSRPPIFPLQPGYFPAAFGYGAASSFLAGSFLRPDYPVFPGMNLLAAAGGSSQGGSLEPI
SPAAYAAYHQQTPNVVLHHSSPGSANGSAGGVVKQESDLGVIMSENQSSSPRSDRSERSPYSD
>gi|241575074|ref|XP_002403447.1| DMRT1, putative [Ixodes scapularis]
MNGHRGQAHDLLRSRSTMRADTHQQQATDLGPRRRHGGSSGNSSGPAPAAARRPTCARCRNHGLRIPVKGHKRYCRFRDCHSPKCSLTVERQKVMAAQVALRRAQAQDEAMGRVPPDEDEEPVLPSDVGGLPGTAPIGALAVSTNSAFRAAAGGKCYSQSARAAGDSVAPICQGECRGHGGRGRRLGDLLGVRIPVRVSGNDDGC
>gi|241575078|ref|XP_002403449.1| doublesex protein, putative [Ixodes
scapularis]
MILMESRAPADGPGRLPSKDGRSAGGTAPGTSSAAASPAKIAGSGARSPQCARCRNHNRKVAVRGHKRYCPYRICVCSKCRLIAERQVVMAQQVALRRAQAQDEASGRAVFEEVDPKSLLDQPPPKDVVKPPPALVTSANSAFAAKGAYRPDAAETGRRVAARKG
>gi|241614034|ref|XP_002406567.1| conserved hypothetical protein [Ixodes
scapularis]
MNGHHATPYHHHHNAAAPGTGGLDANAAAVNSTSADAAARRPKCARCRNHGLRVLVRGHKRRCRYRDCVCPKCKLIAERQRVMAAQVALRRSQAQDEAMGLLPTLQDGCGPSSVSSSDVASLMDVRAHQRHALTNSFRTSVAAAASAGK
>gi|241169514|ref|XP_002410401.1| conserved hypothetical protein [Ixodes
scapularis]
MSVAPSPGELSKRAARVGRSAPPGVRSGKCPEGRLQETLPSWLALDPRVHHH
RSRQESSDGIQAALSTHAHHNMKGERASIKAMSTLVPWRYAGTHRNTVSTQVLVKEQTTKRFSKGRDMKE
GRSCGRAAPASCVGIVRMSSPSQRRASPSPTGSSAAPAGSGGSGARRPKCARCRNHGMISWLKGHKRQC
RFKECACAKCNLIAERQRIMAAQVALKRQQAAEDAIAMGLRAVATGTSLPFLPPGPIFGLPLAAKAMAQAKG
SGPRAKEAAARRAAKEAARTLHRQMRAGRDDGSGDDAAELGERTPHDNSAEAPNTIKASRRISFHDDRAE
HSTPRFAFPSPRPCARRFNGRLGFASPFATVSPVPPRRDGNHIAVGSPLASPHERPPSQRDVAADLPEPP
PPSPDLSVSSSSPAGRSPPPAPSPSGRAASGSPLSTLTRVFPSHRPGFLQLVLRSCDGDLIRAIEQLAHAP
PARSAFRALSPPMAHQGTFFRPAPGPFFRPQGAPFPLAFAAPAYPLLLPCPPGCPQCTGTSSMLAGLAP
SPLRAGHVQQDAWSFLEDTGPDRLKDT
>Rhodnius_RPRC006546__GL546384_1_60593_61439__1_gene_RPRC00654
6
MSSGVGAASQQPMQQQQNPAQPAARTPPNCARCRNHSKTEPLKGHKRFCKY
RTCTCKKCHLTVERQREMAKQTALRRELAQDEARARAGLQPASPPPSATSPP
PPITGQPASHLSTASSLQTTDY
>gi|270011108|gb|EFA07556.1| doublesex [Tribolium castaneum]
MSSDSQDFDSKMDVNASSTSASPRTPPNCARCRNHRLKIALKGHKRYCKYRTCKCEKCRLTTERQRVMAMQTALRRAQAQDEAMLRSGSAVDPAIMQVPLKSPSPIHAIERSLDCDSSASSQCS
NPPPAIRKMTPVPAVPSSTSVNIGTIAQSTDLLEDCQKLLERFKYPWEMMPLMYAILKDARADLEEASRRI
DEGKRVVNEYSRLHNLNMYDGVELRNSTRRDTEILLDFCQRLKDKFQLSWKMISLVDVILKYAKDQDEA
WRQIDEAFLEIRALAAVEAARYTYHHIPYSGLYPNAATAIYPPVYLPSMSMYHPATLLGSVPTSTSPSHS
PPIVPRAIRPSSRA
>gi|270015667|gb|EFA12115.1| hypothetical protein TcasGA2_TC002261
[Tribolium castaneum]
MLSNSRSARVPKCARCRNHGMISTLRGHKKQCIYKNCSCAKCGLIKERQRIMA
AQVALKRQQAAEDAIALHLASAENGTTYDYLPPGRIYGMQVTSPEPEEEKQTAQQETQDEILVSPASIDML
SKLFPNKKRSVLELVLKRCNHDLLKAIEHFNLTNSKSSSSESESSSKEENSSAFKPVEANKPKPFTTPHQ
NSLPLISGSKVFMHSLYPFLPLFNPQPVLPSPVYVPGFCECEQCKHSLYARLEPRQ
>gi|91087421|ref|XP_975675.1| PREDICTED: similar to doublesex-Mab related
99B CG15504-PA [Tribolium castaneum]
MSLPSSGVDMSSLMSQHPVLGAIPPAFFLRASERYQRTPKCARCRNHGVVSALKGHKRYCRWRDCNCAKCTLIAERQRVMAAQVALRRQQAQEENEARELGILFPTPAGVVADTPGVTAPAIPQ
NSDVGISQLMQRNTFTASDSSEPSSPTSKRPRINVEDCSLEGSDSEPEDLKKSRQSSPVPAPAPSAPTP
SPEPQTSPDPDLDVEEDTQSEAPENLSLKKPSSPETPPQPTQNFIPYQQFAFPPFQPQYPAQRSPIDVLMR
VFPGKRRSDVEALLQRCKGDVVQAMEMMVSGSHEDATPPSAFSPLGPPTNFHRFSPSRRFLSAPYAGT
GYLPTVIRPPPDYLSMVGSVHDIYSSDKTSASSPGSNTSSDKTSYSE
