# Supplementary material for: Hemimetabolous insects elucidate the origin of sexual development via alternative splicing
Source: eLife. 2019 Sep 3;8:e47490. doi: 10.7554/eLife.47490 (PMC6721801; doi:10.7554/eLife.47490)
Supplement: Supplementary file 5. [file elife-47490-supp5.rtf]

Supplemental File 5: Queries used in BLASTP searches of arthropod gene models for Transformer and SR family proteins.  

>DrosophilaTra_NP_524114.1
MKMDADSSGTQHRDSRGSRSRSRREREYHGRSSERDSRKKEHKIPYFADEVREQD
RLRRLRQRAHQSTRRTRSRSRSQSSIRESRHRRHRQRSRSRNRNRSRSSERKRRQHSRSRSSERRRRQRS
PHRYNPPPKIINYYVQVPPQDFYGMSGMQQSFGYQRLPRPPPFPPAPYRYRQRPPFIGVPRFGYRNAGRP
PY
>DrosophilaTra2_NP_476764.1
MDREPLSSGRLHCSARYKHKRSASSSSAGTTSSGHKDRRSDYDYCGSRRH
QRSSSRRRSRSRSSSESPPPEPRHRSGRSSRDRERMHKSREHPQASRCIG
VFGLNTNTSQHKVRELFNKYGPIERIQMVIDAQTQRSRGFCFIYFEKLSD
ARAAKDSCSGIEVDGRRIRVDFSITQRAHTPTPGVYLGRQPRGKAPRSFS
PRRGRRVYHDRSASPYDNYRDRYDYRNDRYDRNLRRSPSRNRYTRNRSYS
RSRSPQLRRTSSRY
>DrosophilaSFRS_NP_996288.1
MWHEARKQERKIRGLIVDYRKRAERRQYFYDKIQADPTQFLQLHGRRSKIYLDPA
VAAAGDGAAIIVPWQGQQDNLIDRFDVRAHLDHIPPVNKTNAEGADGDSELTLEERQLNYERYRILAQNDFLNVSEDKFL
HQLYLEEQFGANAQLEAERNLAKKKQKTGGATIGYSYEDTGDGATIGPQPFGAASSTVIGGAAAVAKGEKGDGSEESDSD
IDMDVSIDIGKLDTSQAHELNACGRNYGMKSNDFFSHLTKDADEADALRIAREEEQEKMLLSGRKSRRERRAQKERRIAN
RPFSPPSYAAKEDKDKKGGGKGGEKEKEEDSESRSPSPAEAGPEKITYITSFGGEDEMQPHSKITISLT
>Drososphila_Pinin_NP_649934.1
MVNDSGLSTVDDLEQKLNSAKQSLVILNENIRRIAGRVPKESLQRSEKFKYTQDG
KKNEHNGDRPFPRNATPGGVFKDKRRMYESKNPISRFPIEENEGRPPRINSRVIREMPTKKEIVEAQGTDSESRARNRRM
FGSLLGTLQKFCQEESRLKSKEDKKAEIDRKVEKQELQERAMLRKQRETLFLDRKKKQFEIRRLEYKMARMKDFKVWEAT
MLNAKNNIRTKTKPHLFFRPKVHSPRTEKLLSKSKSEADVFIEFRREELEVELKNLENMNFGKMEDDTAIDESFYEEPDD
EEQLDKCK
>DaphniaTra_AGM48362.1
MMRPRSRSRAGQSSNFQRSRSRSPFYNREKHNRFEGKPFRGNSSYHQHGRPDEVR
RREEYNRPTNISPRRSPGPYKHRSRSHSPHPRVHHQHQQNTRKGDFNNDIRRDHRSPVHYDTMYRRSPTP
QRLTTEPIPKDRSIFRGPEGTVIDLNELKKITVDIRRNLARGTIPASHSPLRYAFNPSDVVLVRRPGEGS
RQIFDREELKPRPAEERVIKLAADLPDRLEHQQRSPSRSHDPFAGTSGSSFQQESRSVDGDGEGDLRFRL
MEKKSSEENKERMNADPNFVPQGRYYYEHDNRERYMNRGGRGFAFRGNGRGNYHLNQQGGSSVSGYRGNF
RGGGGGNGGGQYRESYRGSYRGKMRSPNWQHDLYDTAPVDDGKPSSTTQI
>DaphniaTra2_EFX90042.1
MDSPRRKDSPSGNYRHHKDSGRSRSPGHRHQARRSRSYDEDRDRRSHHDRRDRGD
RGGDRGGDRYHSRSRSPMSGRKRHNNMHGGSHRGGGGGGGGGGGGGGGGGGGGPPDNPEPTRCLGVFGMG
LYTTETELQHVFAKYGPLEKVQVVKDAKTGRSRGFAFVYFESLEDAKLAKEQCTGLEIDGRRIRVDYSIT
QRPHTPTPGIYMGRPTITSGRYDKYDRNERGYRRSPSPYYEKNYRSSRRGYERSRSRSYSPRRY
>DaphniaPinin_EFX77618.1
MAVAVENFQAELEKARETLNKVDENIKKITGRDPTEARNARFQSQVGNNEGRENA
QARNRIPPRFQGRLSSRDEQEKRHDGPTHTKRIVREGNDGRPTPLVAIGNPKGWRNDDYEEGELPRKGAI
HSSITAPNADRKTREEALAQQSKDVKVKDRNRRMFGSLLGTLAKFRMEESSQKDKEEKRARIEQRLEETA
RQEKESLRKERNELFMERKRQQAHVRRLEWKMHRLREHDEWATRQQPLMKFIKLTTTPPLYYKPKVWTPK
TEELLKESQRKLKEMLETRRKELEEELLRVEEKAIARLERVRERGYRVAPASEQDAELEELEAEIEMYAL
ALGEEKEEDMELHPE
KVSQGPLTFPKHDPDEPMDSA
>DaphSFRS_EFX70995.1
MVDHRRRAERRREYYEKTKGDPAQFLQVHGRPMKIHVDPAIAAIADSPATMMPWQ
GQPEILIDRFDVRAHLDVIPCRPQKDDSDTDQDPGEEVWEERQASYERYRILVQNEFIGVSEEKFLHQIL
LEEQFGPVQKLGEEEKKKMLAPKKAAIGFTYDDSTPSSSAAASTSHNIPVVPPEATEEEESDSDIDLDLS
IAVDKLTTEQMHEVNKCALHYGLGRQDFMSLLTRDLDEQESLRIAKEEEQERAMYSGRKARKERRAFREQ
KLQALLMNRCLSPPSYATRASPTYDGQLRRSKSVSKSRSPSPFEGGIKFITSFGDED
>RoachTra
MRSRSPSRRSTPPPPRISRRSPGAFRQRGKSPERRRRMVPPAGVPKGAYVQAHIR
PRRSRSPVQREPKGRSFSPSKRKLSPKRDRERIRQRDPERERMRTRDPDRERGRS
RERPPMREREPSHMDLVPPVKRTRSKSPDGPMGSRLSNYAGSVCGPSDLSPARSY
QGPPGMYPQGPRSDREETNRNVVSLSERFHSSGPGSYKKESEHPVFRGPEGSGFD
LTDLKKITMVIRRNPPVEMVPIERNILNPEDVVLKRRPGM
>Blattella_BGER028045-PA
ISGVIRVLEECLTVAATGHEAVLAHLSATSLVIHTLDPVHTALVENTMDTAIDAK
NSTEVIHAAPCPPDGATWGAGYVKININCAFVFEFQDNPQPSRCLGIFGLSIYTT
EQQLHHIMSKYGPVERVQVVIDAKTGRSRGFSFVYFESAEDAKVAKEQCTGMEID
GRRIRVDFSITQRAHTPTPGIYMGKPTYHSEGRGQWGGRQKG
>BlattellaPinin_BGER018032-PA
LYSIIVIMATEIMKSFGALQAELERAKDNLKGVDENIRKLIGRDPSEGQQRPGGL
KRPAAQPEEFRGRGRGFGLGRGRRDPGGRPEEEEPVQVVKRRPGDTRTVFSRLSG
PPRLKREGDSGDEEDVANKPAISSRVIATPKELPSRQEAMAAQSTDERSKARNRR
MFGALLGTLQKFRQEETRMRDKEEKRAKVEKKLEENAKREKEELKKERQELFQER
KRKQAEIKKIELKLLRMKEQEEWEKMIEKKRQELLSELEAIEKRGRWRQGLGDSE
GGIGQVGNNDLQGDMEMMEVGDDEHRLEVNEESPEAGKDVVNRAGEEDVRGSMPV
VNQSADNIEVIEPRIDERNIKKEIMERMKGSSGEGGEEDDDDDDEDEEDDDDDDD
NRNKESSRNSSEKEQESSKGKFKEGVIVKAEPINSEST
>Blattella_SFRS_BGER003059-PA
MPWQGRQDVLIDRFDVRAHLDYIPENSLQTDSASNDELSREDRLANYERYRIIVQ
NDFLGIGEEKFLHQLHIEEQFGPVSRTNEVDKRKREKGATGAAIPYSYEDTTSGP
GATEEEEAEGDEEKDEEEEDDDDSDIDFDLCVDVSQIGPQQAHEMNVCGAAYGMS
GNDFYTFLTRDIEEKESLRQAREQEEEKAMYSVSI
