# Supplementary material for: Hemimetabolous insects elucidate the origin of sexual development via alternative splicing
Source: eLife. 2019 Sep 3;8:e47490. doi: 10.7554/eLife.47490 (PMC6721801; doi:10.7554/eLife.47490)
Supplement: Transparent reporting form [file elife-47490-transrepform.pdf]

eLIFE
1st Floor
24 Hills Road
Cambridge CB2 1JP, UK
P 01223 855340
W elifesciences.org
T @elife

eLife's transparent reporting form

We encourage authors to provide detailed information *within their submission* to facilitate the interpretation and replication of experiments. Authors can upload supporting documentation to indicate the use of appropriate reporting guidelines for health-related research (see EQUATOR Network), life science research (see the BioSharing Information Resource), or the ARRIVE guidelines for reporting work involving animal research. Where applicable, authors should refer to any relevant reporting standards documents in this form.

If you have any questions, please consult our Journal Policies and/or contact us: editorial@elifesciences.org.

Sample-size estimation

- You should state whether an appropriate sample size was computed when the study was being designed
- You should state the statistical method of sample size computation and any required assumptions
- If no explicit power analysis was used, you should describe how you decided what sample (replicate) size (number) to use

Please outline where this information can be found within the submission (e.g., sections or figure legends), or explain why this information doesn't apply to your submission:

Sample size was dictated by the number of roaches we could inject at any given time point (a function of how many individuals we had of the correct stage and sex). This is mentioned in **the Materials and Methods section**.

Replicates

- You should report how often each experiment was performed
- You should include a definition of biological versus technical replication
- The data obtained should be provided and sufficient information should be provided to indicate the number of independent biological and/or technical replicates
- If you encountered any outliers, you should describe how these were handled
- Criteria for exclusion/inclusion of data should be clearly stated
- High-throughput sequence data should be uploaded before submission, with a private link for reviewers provided (these are available from both GEO and ArrayExpress)

Please outline where this information can be found within the submission (e.g., sections or figure legends), or explain why this information doesn't apply to your submission:

eLife Sciences Publications, Ltd is a limited liability non-profit non-stock corporation incorporated in the State of Delaware, USA, with company number 5030732, and is registered in the UK with company number FC030576 and branch number BR015634 at the address 1st Floor, 24 Hills Road, Cambridge CB2 1JP | August 2014

For *Rhodnius prolixus* and *Blattella germanica* RACE, Illumina sequencing, and PacBio Isosequencing: All libraries were created from RNA collected from reproductive tract or gonad of single males or females. We used separate RNA samples/individuals for RACE, Illumina sequencing, and PacBio Isosequencing. **This information is contained in the Materials and Methods section.**

For *Pediculus humanus* RACE and Illumina sequencing, a single sample/library was created from pools of 6-10 whole adult males or females. **This information is also included in Materials and Methods.**

qPCR was conducted on two to three biological replicates (individuals) per sex in two species: *B. germanica* and *R. prolixus*. Per individual, we ran three technical replicates. A technical replicate was defined as a duplicate reaction with identical template, reagents, and cycling conditions. **This information is contained in the Materials and Methods section.** All *B. germanica* qPCRs were ran with three individuals per sex per trial. qPCR for *R. prolixus dsx* was conducted with two biological individuals per sex. This information is also included **in Materials and Methods.**

For RNAi experiments performed on *B. germanica* nymphs, sample size for morphological phenotypes recorded is **reported in the Results section**. All morphological phenotypes were observed in a minimum of 2 replicated injections. For oligosaccharide experiments, **sample size is reported both in the Results section and in the caption of Figure 4-figure supplement 1**. For parental RNAi experiments, **sample size is recorded in Figure 5 and in Figure 7-figure supplement 4.**

Statistical reporting

- Statistical analysis methods should be described and justified
- Raw data should be presented in figures whenever informative to do so (typically when N per group is less than 10)
- For each experiment, you should identify the statistical tests used, exact values of N, definitions of center, methods of multiple test correction, and dispersion and precision measures (e.g., mean, median, SD, SEM, confidence intervals; and, for the major substantive results, a measure of effect size (e.g., Pearson's r, Cohen's d)
- Report exact p-values wherever possible alongside the summary statistics and 95% confidence intervals. These should be reported for all key questions and not only when the p-value is less than 0.05.

Please outline where this information can be found within the submission (e.g., sections or figure legends), or explain why this information doesn't apply to your submission:

For behavioral phenotypes, sample size and statistical test used are reported in the **Results section and in the caption of Figure 4**. For the CHC analysis, sample size and statistical test are reported in the **caption of Figure 4-figure supplement 2.** For parental dsTra, sample size and statistical test are reported in the **caption of Figure 5.**

(For large datasets, or papers with a very large number of statistical tests, you may upload a single table file with tests, Ns, etc., with reference to sections in the manuscript.)

Group allocation

- Indicate how samples were allocated into experimental groups (in the case of clinical studies, please specify allocation to treatment method); if randomization was used, please also state if restricted randomization was applied
- Indicate if masking was used during group allocation, data collection and/or data analysis

Please outline where this information can be found within the submission (e.g., sections or figure legends), or explain why this information doesn't apply to your submission:

Roaches were randomly assigned into control or experimental treatment for RNAi.

Additional data files ("source data")

- We encourage you to upload relevant additional data files, such as numerical data that are represented as a graph in a figure, or as a summary table
- Where provided, these should be in the most useful format, and they can be uploaded as "Source data" files linked to a main figure or table
- Include model definition files including the full list of parameters used
- Include code used for data analysis (e.g., R, MatLab)
- Avoid stating that data files are "available upon request"

Please indicate the figures or tables for which source data files have been provided:

eLife Sciences Publications, Ltd is a limited liability non-profit non-stock corporation incorporated in the State of Delaware, USA, with company number 5030732, and is registered in the UK with company number FC030576 and branch number BR015634 at the address 1st Floor, 24 Hills Road, Cambridge CB2 1JP | August 2014
